# Interactive real-time mapping and ablation of the pulmonary veins and cavotricuspid isthmus in an ovine model with an externally-irrigated MRI-compatible ablation catheter

**DOI:** 10.1186/1532-429X-14-S1-O103

**Published:** 2012-02-01

**Authors:** Anand N Ganesan, Suchi Grover, Rajiv Mahajan, Sachin Nayyar, Dan Sunnarborg, Tom Lloyd, Prashanthan Sanders, Joseph Selvanayagam

**Affiliations:** 1 Centre for Heart Rhythm Disorders, University of Adelaide, Adelaide, SA, Australia; 2 Cardiology, Flinders University of South Australia, Adelaide, SA, Australia; 3 Imricor Medical Systems, Burnsville, MN, USA

## Summary

In recent years MRI-guided interventional electrophysiology (EP) has rapidly emerged as a promising alternative to conventional X-ray guided ablation. We present a novel MRI-compatible electrophysiologic ablation and recording system, performing realistic biatrial simulated procedures.

## Background

To date, preclinical studies evaluating MRI-guided ablation have used non-irrigated catheters limited to test lesions in the right heart. Modern complex EP procedures require externally irrigated catheters to produce adequate lesion depth. We present the first feasibility study of an externally-irrigated MRI-compatible ablation catheter, and integrated EP pacing and recording system. We demonstrate the feasibility of pulmonary vein (PV) and cavotricuspid isthmus (CTI) ablation, the targets of ablation in atrial fibrillation and typical atrial flutter respectively.

## Methods

An externally irrigated MRI-compatible ablation and diagnostic electrophysiology catheters and integrated EP recording system (Imricor Medical Systems, Burnsville, MN) were tested in n=11 sheep, in a 1.5T MRI scanner. The diagnostic catheters were placed in the coronary sinus, and right ventricular apex. Power controlled (40W, 120 second duration) lesions were formed at the pulmonary veins and cavotricuspid isthmus with the ablation catheter. Real-time intracardiac electrograms were recorded during MR imaging. Steady state free precession (SSFP) non-breath-hold images were repeatedly acquired to guide the catheter to the target region using passive catheter tracking. Markers in the catheter were used to ensure adequate visualization of the catheter. Lesion visualization was performed using both non-contrast (T2-weighted turbo spin echo pulse sequence) and gadolinium-DTPA enhanced T1-weighted imaging (inversion-recovery gradient echo pulse sequence).

## Results

The catheters were able to be visualised and navigated throughout the heart. An average of 8±2.5 lesions were formed near the ostia of the pulmonary veins, and 6.5±1.3 lesions were formed at the cavotricuspid isthmus. The electrophysiological endpoint of bidirectional block was achieved in all cases. Mean procedure duration was 150±55 minutes. Histologic examination demonstrated transmurality of lesion formation at both the pulmonary vein ostia, and cavotricuspid isthmus. Lesion visualisation with both T2W imaging and contrast-enhanced imaging correlated with sites of injury at autopsy (see image). Non-contrast agent-enhanced techniques were suitable for repetitive lesion visualization during interventions, thus allowing for intra-procedural monitoring of ablation success.

## Conclusions

These data represent the most realistic simulated interventional MRI-guided electrophysiology procedures in the published literature, demonstrating the use of multiple catheters, an integrated EP pacing and recording system, and the use of an externally-irrigated ablation catheter. For the first time, the feasibility of CMR guided pulmonary vein ablation is shown, opening the possibility of MR-guided complex electrophysiology procedures, including pulmonary vein isolation for atrial fibrillation.

## Funding

Dr Ganesan is funded by a Cardiovascular Lipid award.

**Figure 1:**
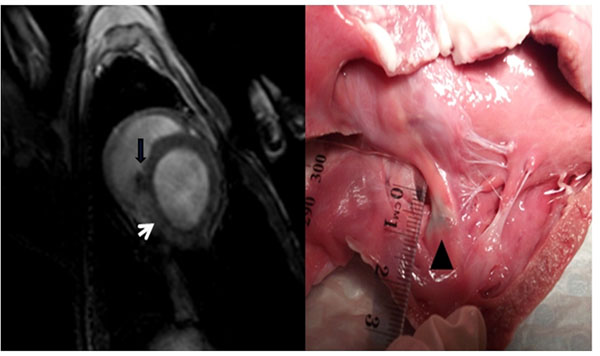
(Left panel) Late gadolinium enhancement image at caovtricuspid isthmus level, showing myocardial injury (white arrow) with T2W turbo spin sequence. Ablation catheter is seen (black arrow). (Right panel) Macroscopic lesion at CTI (black triangle).

